# PD22 Economic Evaluation Of Percutaneous Nephrolithotomy, Flexible Ureterorenoscopy, And Extracorporeal Shockwave Lithotripsy For Lower Pole Kidney Stones

**DOI:** 10.1017/S0266462325101578

**Published:** 2025-12-29

**Authors:** Rodolfo Hernández, Oliver Wiseman, Daron Smith, Kathryn Starr, Lorna Aucott, Ruth Thomas, Steven MacLennan, Charles Terry Clark, Graeme MacLennan, Victoria Bell, Seonaidh Cotton, Sam McClinton

## Abstract

**Introduction:**

Renal stone disease is common, affecting mainly working-age adults, with a lifetime prevalence of 10 percent across the world. Approximately 50 percent of people with renal stones will experience symptoms and 25 percent will require treatment. This study assessed the cost effectiveness of flexible ureterorenoscopy (FURS), compared with extracorporeal shockwave lithotripsy (ESWL) (PUrE-RCT1) and percutaneous nephrolithotomy (PCNL) (PUrE-RCT2) for treating stones in the lower pole of the kidney.

**Methods:**

The UK PUrE studies were pragmatic, multicenter, open-label superiority randomized controlled trials recruiting participants with lower pole stones sized 10 mm or less (n=466; PUrE-RCT1), and more than 10 but less than 25 mm (n=159; PUrE-RCT2). Mean costs to the health service and quality-adjusted life years (QALYs) were estimated using generalized linear models by intention-to-treat 12 months after randomization. A Markov model used trial data, combined with longer term data from the literature, for a five-year extrapolation based on the expected recurrence due to residual fragments. Uncertainty around results was characterized by conducting probabilistic and deterministic sensitivity analyses.

**Results:**

For PUrE-RCT 1, FURS was, on average, more costly (GBP1,138 [USD1,536], 95% confidence interval [CI]: GBP646 [USD872], GBP1631 [USD2,202]) and produced 0.017 (95% CI: −0.008, 0.043) additional QALYs with an incremental cost-effectiveness ratio (ICER) of GBP65,163 (USD87,970) per QALY gained. However, ESWL had a 99 percent chance of being cost effective at a GBP20,000 (USD27,000) cost per QALY threshold. For PUrE-RCT2, FURS was, on average, more costly (GBP733 [USD990], 95% CI: −GBP508 [−USD686], GBP1,973 [USD2,664]) when a micro-costing approach was used and produced fewer QALYs (−0.001, 95% CI: −0.044, 0.042). PCNL had an 87 percent chance of being cost effective at a GBP20,000 (USD27,000) threshold value. However, FURS was cost effective when the Healthcare Resource Group (HRG) costing approach was used.

**Conclusions:**

PUrE-RCT1 found that ESWL was cost effective, compared with FURS, with no meaningful difference in QALYs gained, even though stone-free rates were higher after FURS. In PUrE-RCT2, PCNL was cost effective compared with FURS, but the results were sensitive to the costing approach used. Higher transparency in the methods used to derive HRG costing is needed.

